# Advances in the Aetiology and Treatment of Anorexia Nervosa

**DOI:** 10.3390/jcm11206068

**Published:** 2022-10-14

**Authors:** Andrea Phillipou

**Affiliations:** 1 Centre for Mental Health, Faculty of Health, Arts & Design, Swinburne University of Technology, Hawthorn, VIC 3122, Australia; andreaphillipou@swin.edu.au; 2 Department of Mental Health, St Vincent’s Hospital, Fitzroy, VIC 3065, Australia; 3 Department of Mental Health, Austin Health, Heidelberg, VIC 3084, Australia

Anorexia nervosa (AN) is a complex psychiatric disorder. The hallmark features of AN include significant body image disturbances which lead to reduced energy intake, and consequently, very low body weight [1]. As such, although AN is fundamentally a mental illness, the psychological and behavioural features result in a number of physical consequences due to the effects of starvation [2]. Although AN is associated with significant psychological and physical morbidity, and mortality, the illness remains poorly understood. Consequently, treatment outcomes for individuals with AN are generally poor with less than 63% of individuals reported to recover from the illness in the long-term [3]. In order to improve outcomes for individuals with AN, a better understanding of the illness is required so that more effective treatments can be developed.

In this Special Issue, a range of topics are presented to advance our understanding of the underlying mechanisms potentially involved in AN. Miles, Nedeljkovic, and Phillipou [4] present the findings of a sample of individuals with AN compared to a community sample of individuals without a history of an eating disorder. In this study, individuals with AN reported poorer cognitive flexibility and increased clinical perfectionism, relative to the control group. The authors also found that clinical perfectionism, but not cognitive flexibility, could identify AN from control participants with high sensitivity and specificity, suggesting that clinical perfectionism may be a key feature that underlies AN.

Huang and Foldi [5], on the other hand, present a review on the activity-based anorexia (ABA) model, and how this model could be used to learn more about the neural underpinnings of cognitive inflexibility in humans with AN. The authors describe the overlapping features of dysregulated serotonin and dopamine signalling, and disrupted prefrontal circuitry, described in both the cognitive inflexibility and AN literature, and suggest that models such as ABA have the potential to be used to elucidate these features and inform therapeutics for AN.

Frintrop, Trinh, Seitz, and Kipp [6] also provide a review of the literature pertaining to the underlying neurobiology of AN, but with a focus on the potential role of glial cells in the illness. The authors describe the role of glial cells in eating behaviour, and how they may be involved in the neuropsychological progression of AN.

Finally, West et al. [7] describe the range of gastrointestinal disturbances experienced by individuals with AN, and the scant literature regarding management and treatment of these issues by dietitians. Their study revealed that dietitians with a greater understanding of gastrointestinal disturbances in AN recognised its impact on the individual’s quality of life and were more likely to screen patients for such disturbances. To optimise treatment outcomes for individuals with AN, the authors concluded that greater knowledge and confidence in addressing gastrointestinal disturbances is required.

The novel publications presented in this Special Issue cover a broad range of topics pertinent to our understanding of the aetiology and treatment of AN. This research contributes to our growing understanding of the illness, and as with all research, brings us one step closer to improving outcomes for our patients.
